# Effects of alternate-day fasting on body weight and dyslipidaemia in patients with non-alcoholic fatty liver disease: a randomised controlled trial

**DOI:** 10.1186/s12876-019-1132-8

**Published:** 2019-12-18

**Authors:** Hua Cai, Yue-Lan Qin, Ze-Ya Shi, Jin-Hui Chen, Min-Jie Zeng, Wei Zhou, Ru-Qun Chen, Zhi-Yuan Chen

**Affiliations:** Department of Gastroenterology, Hunan Provincial People’ Hospital, The First Affiliated Hospital of Human Normal University, Jiefang Road West, Changsha, 410005 Hunan China

**Keywords:** Alternate-day fasting, Weight loss, Dyslipidaemia, Non-alcoholic fatty liver disease

## Abstract

**Background:**

Alternate-day fasting (ADF) is a novel diet therapy that may achieve reduction in body weight and improvement of dyslipidaemia, but the impact of this diet on patients with non-alcoholic fatty liver disease (NAFLD) remains unknown. The aim of this study was to evaluate the effects of ADF on the body weight and lipid profile of individuals with NAFLD.

**Methods:**

NAFLD patients (*n* = 271) were randomised to the ADF group, time-restricted feeding (TRF) group, or the control group and subjected to the respective diet for 12 weeks. Anthropometric measurements (body weight, fat mass/fat-free mass) were performed, and plasma lipids were analysed enzymatically.

**Results:**

Within 4 weeks, the body weight decreased significantly (*P* < 0.001) in the ADF group by 4.56 ± 0.41 kg (6.1 ± 0.5%) and the TRF group by 3.62 ± 0.65 kg (4.83 ± 0.9%) compared to the control group, and it decreased even more after 12 weeks in both groups (ADF: − 4.04 ± 0.54 kg, 5.4 ± 0.7%; TRF: − 3.25 ± 0.67 kg, 4.3 ± 0.9%). Fat mass was significantly reduced by ADF (− 3.49 ± 0.37 kg; 11 ± 1.2%) and TRF (− 2.91 ± 0.41 kg; 9.6 ± 1.3%), with ADF leading to a further reduction in fat mass after 12 weeks (− 3.48 ± 0.38 kg; 11 ± 1.2%). Total cholesterol was significantly decreased at both time points in the ADF group (− 0.91 ± 0.07 mmol/L; 18.5 ± 1.5%) compared to the control and TRF groups. Both ADF (− 0.64 ± 0.06 mmol/L; 25 ± 1.9%) and TRF (0.58 ± 0.07 mmol/L; 20 ± 1.7%) achieved a significant reduction in serum triglycerides (*P* < 0.001) after 12 weeks. Changes in fat free mass, HDL, LDL, fasting insulin, glucose, liver stiffness, and systolic or diastolic blood pressure did not differ between the groups.

**Conclusions:**

ADF appears to be an effective diet therapy for individuals with NAFLD that can achieve weight loss and improvement of dyslipidaemia within a relatively short period of time (4 to 12 weeks). Potential preventive effects of ADF on cardiovascular disease need to be confirmed by future investigations.

**Trial registration:**

ChiCTR1900024411, this trial was retrospectively registered on July 10, 2019.

## Background

Non-alcoholic fatty liver disease (NAFLD) is a spectrum of liver disease characterised by steatosis, a deposition of ectopic fat in the liver, that in contrast to alcoholic fatty liver disease cannot be explained by excessive alcohol consumption. The disease is strongly associated with obesity, insulin resistance (IR)/type 2 diabetes mellitus (T2DM), and the metabolic syndrome which may eventually lead to complications such as cirrhosis, liver cancer, liver failure, or cardiovascular disease [1–3]. Lifestyle modifications including diet and exercise are considered the initial treatment of choice with the aim to reduce weight gain and the risk of developing insulin resistance and metabolic syndrome [4, 5].

Intermittent fasting (IF) is a novel weight loss regimen that has been steadily growing in popularity over the past decade [6, 7]. Time-restricted feeding (TRF) is a form of IF that allows specific time windows for food consumption throughout the day. The most popular TRF feeding schedule is 16:8, meaning that an 8-h window for food intake is followed by a 16-h window of fasting [8–10]. In previous studies, significant improvements in body mass and serum lipid profiles have been achieved with TRF. This type of diet therefore has the potential to be a cost-effective intervention in NAFLD patients [11]. Alternate-day fasting (ADF) is an innovative form of IF that involves alternation between a feed day with ad libitum food intake and a fast-day with an energy restriction of 75% [12, 13]. Several studies have demonstrated that ADF administration for 4–12 weeks may effectively reduce body weight and visceral fat mass [12, 14]. ADF appears to achieve a higher weight loss than other forms of IF [15]. In addition to these favourable effects of ADF on body composition, beneficial outcomes on blood lipids (decreases in LDL cholesterol and triglycerides, increases in LDL particle size) have been reported [16, 17]. Although evidence from clinical trials is limited, preliminary findings indicate that ADF could be an effective therapeutic strategy for NAFLD patients.

Therefore, the goal of this study was to compare the effects of ADF and TRF on body weight, body composition, and dyslipidaemia in individuals with NAFLD in a 12-week randomised controlled feeding trial. We hypothesised that both ADF and TRF are safe and tolerable diets that achieve a higher weight loss and favourable effects on body composition and plasma lipid profile compared to the control diet, thereby decreasing the risk for cardiovascular disease in NAFLD patients.

## Methods

### Subjects

This study was a randomized clinical trial, which was designed according to CONSORT guideline. Subjects were recruited from the Changsha area in China through advertisements in the vicinity of the Hunan Provincial People’s Hospital. A total of 285 adults with NAFLD that met the initial eligibility criteria were invited for a screening visit. NAFLD diagnosis was confirmed by abdominal ultrasound with liver stiffness > 9.6 kpa [18, 19]. Inclusion criteria were as follows: BMI > 24 kg/m^2^; age between 18 and 65 years; stable body weight for 3 months prior to the beginning of the study (< 5 kg weight loss or weight gain). Exclusion criteria were cardiovascular disease, uncontrolled hypertension, chronic inflammatory diseases, chronic infections, cancer, taking weight loss, lipid-, or glucose-lowering medications, and a history of bariatric surgery. Pregnant women and those planning a pregnancy or lactating were also excluded. All subjects provided written informed consent to participate in this study. The protocol was approved by the ethics committee of Hunan Provincial People’s Hospital (2018–37), and all research participants gave their written informed consent to participate in the trial.

### Experimental design

**Fig. 1:**
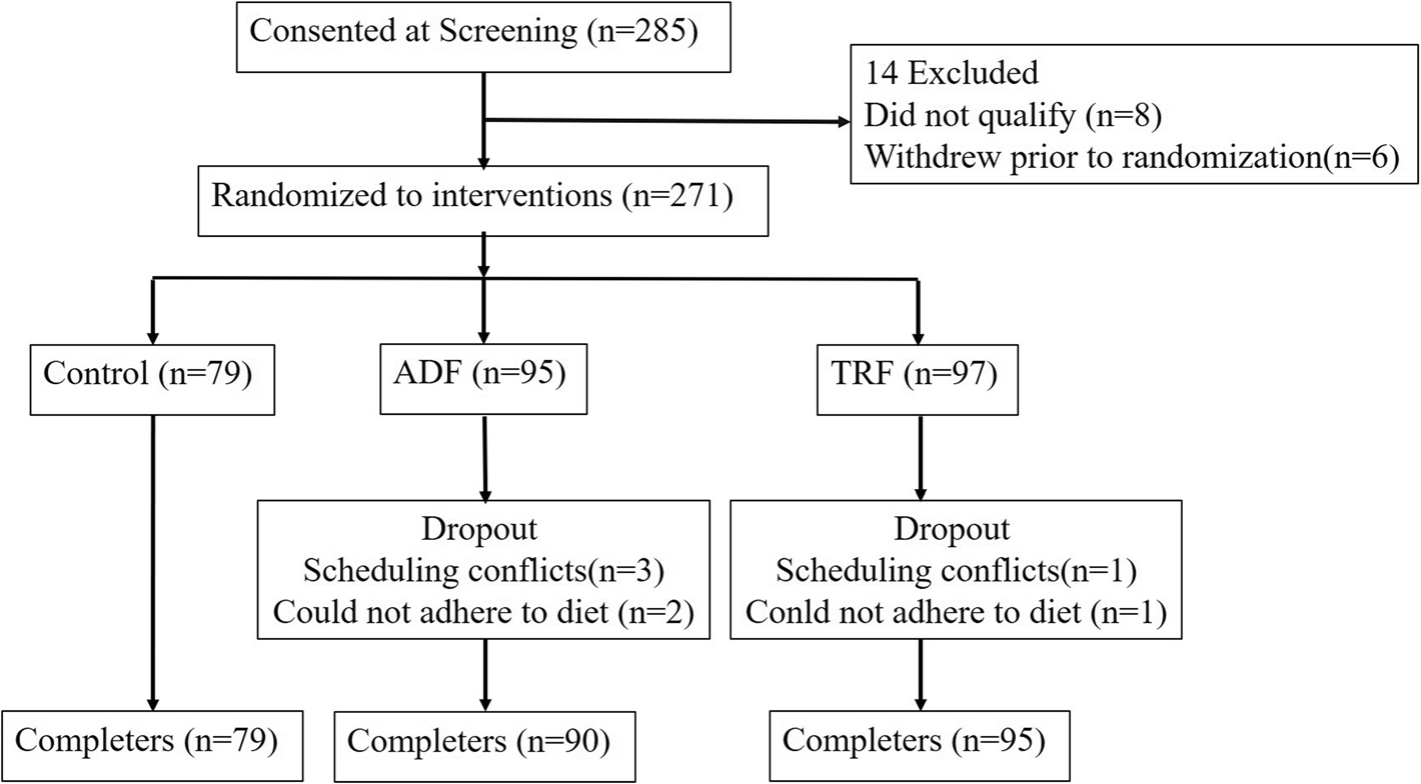
Flow chart of the study protocol. ADF: alternate-day fasting; TRF: time-restricted feeding

Block randomization was performed with a computer-generated random number sequence. An independent statistician generated the allocation sequence, and the study coordinator assigned the subjects to controlled dietary interventions trial for 12 weeks as the subjects enrolled. Subjects were randomised using the stratified random sample to groups: 1) control (*n* = 79); 2) ADF (*n* = 95); or 3) TRF (*n* = 97) (Fig. 1).

### Dietary intervention

During the dietary intervention period, individuals in the ADF group consumed 25% of their baseline energy needs through meals prepared in the metabolic kitchen of Hunan Provincial People’s Hospital, Changsha on the fast day (24 h), and then ate ad libitum at home on the feed day (24 h). Fast day meals were provided as a 3-day rotating menu, and were formulated based on the American Heart Association (AHA) guidelines (30% kcal from fat, 15% kcal from protein, 55% kcal from carbohydrate) [16, 20]. Energy needs for each subject were determined with the Mifflin equation. The feed and fast days began at midnight, and all fast day meals were consumed between 12.00 p.m. and 2.00 p.m. to ensure that all subjects were undergoing the same duration of fasting. Consumption of energy-free beverages, tea, coffee, and sugar-free gum was permitted and participants were encouraged to drink plenty of water. TRF subjects were provided with a meal within an 8-h window and asked to refrain from consumption of all food or beverages that included energy for the remaining 16 h. There were no additional instructions or recommendations on the amount or type of food consumed during the 8-h window. The timing of the feeding window during the day could be freely chosen to accommodate lifestyle habits of the participants. Subjects in the control group consumed 80% of their energy needs every day without any recommendations for or restrictions on their usual lifestyle patterns.

### Outcome measures

Anthropometric measurements were performed at the beginning of each week throughout the study period. Body weight was measured to the nearest 0.1 kg using a calibrated digital scale with the subjects wearing shoes and light clothing. Body height was determined to the nearest 0.1 cm using a wall-mounted stadiometer. Waist circumference was measured to the nearest 0.1 cm using a flexible tape placed midway between the lower costal margin and the super iliac crest during expiration. Body composition (fat mass and fat-free mass) was assessed by dual x-ray absorptiometry (DXA, Discovery-W version 12.6, Hologic, Bedford, MA, USA).

The value of liver stiffness was measured by Fibrocan (Echosens Corp., Paris, France). A total of 10 measurements were carried out, and liver stiffness value was record only if the interquartile range did not exceed 40% in any of the measurements. The results were expressed in kilopascals. The median value was taken as representative [18, 19].

Fasting blood samples were collected at 6.00 a.m. at baseline and at week 4 and week 12 after initiation of the intervention. Participants were instructed to avoid exercise, alcohol, and coffee for 24 h before each visit. The collected blood was centrifuged for 10 min at 520×g at 4 °C to separate plasma from red blood cells and was stored at − 80 °C until analysis. Plasma total cholesterol, direct LDL cholesterol, HDL cholesterol, and triglyceride concentrations were measured in duplicate with enzymatic kits (Biovision, Mountainview, CA, USA). LDL particle size was determined by linear polyacrylamide gel electrophoresis (Quantimetrix Lipoprint System, Redondo Beach, CA, USA).

A validated visual analog scale (VAS) was used to measure hunger, fullness, and satisfaction with the study diet [21]. The scale was completed at baseline and at week 4 and week 12. The VAS consisted of 100-mm lines and subjects were asked to place a vertical mark across the line corresponding best to their feelings of hunger, satisfaction, and fullness, with the scale ranging from 0 (not at all) to 100 (extremely). Quantification was performed by measuring the distance between the left end of the line and the vertical mark.

### Statistical analysis

All statistical analyses were performed using the Statistical Package for the Social Sciences software (SPSS version 19.0, IBM, Armonk, NY, USA). Numerical data were reported as mean ± standard deviation and/or range. Continuous variables were compared using one-way analysis of variance (ANOVA) and categorical variables using Fisher’s exact or Pearson chi-square tests. A *P*-value < 0.05 was considered statistically significant.

## Results

### Subject characteristics

Out of 285 randomised subjects, 14 withdrew before the intervention, leaving 95 subjects in the ADF group, 97 in the TRF group, and 79 in the control group. Eight weeks into the trial, a further 5 subjects dropped out of the ADF group and 2 dropped out of the TRF group due to scheduling conflicts and personal reasons. Therefore, 90 subjects in the ADF group, 95 in the TRF group, and 79 in the control group completed the study (Fig. 1). Baseline characteristics of the subjects are presented in Table 1. There were no statistically significant differences in age, sex, body weight, body composition, height, or BMI between the groups at the beginning of the study.

**Table 1:** Subject characteristics at baseline

Characteristic	Control(*n* = 79)	ADF(*n* = 90)	TRF(*n* = 95)	*P*-value
Age (y)	34.54 ± 6.96	35.50 ± 4.417	33.56 ± 6.23	0.085
Sex (M/F)	23/79	35/60	29/66	0.089
Body weight (kg)	72.94 ± 8.00	75.32 ± 8.53	74.98 ± 8.02	0.396
Height (cm)	166.39 ± 7.59	169.73 ± 6.91	167.30 ± 7.45	0.122
BMI	26.34 ± 2.73	26.12 ± 2.21	26.76 ± 1.59	0.510
Fat mass (kg)	29.06 ± 3.64	30.58 ± 3.95	30.27 ± 3.23	0.248
Lean mass (kg)	43.65 ± 3.95	44.69 ± 4.57	44.54 ± 4.08	0.347
Waist circumference	92.59 ± 4.98	92.07 ± 5.29	91.54 ± 4.43	0.367
Cholesterol (mg/dL)	4.88 ± 1.38	4.87 ± 1.02	4.53 ± 1.53	0.142
Total Direct HDL(mg/dL)	1.16 ± 0.50	1.23 ± 0.43	1.16 ± 0.45	0.487
LDL (mg/dL)	2.55 ± 0.79	2.82 ± 0.85	2.73 ± 0.88	0.101
Triglycerides (mg/dL)	2.65 ± 1.69	2.80 ± 1.90	2.90 ± 1.75	0.660
Glucose (mg/dL)	5.09 ± 0.90	5.21 ± 0.86	5.12 ± 0.82	0.639
Liver stiffness (kpa)	18.79 ± 3.55	18.48 ± 4.82	18.71 ± 3.75	0.880

No adverse events were associated with the intervention during the 12-week study period. Energy intake, hunger, and fullness are reported in Table 2. There were no differences in the energy intake between the groups (on feed day) at baseline. Between week 4 and week 12 of the study, energy intake remained constant in the ADF group on both the feed and fast days. Fullness levels increased from week 4 to week 12 in the ADF and TRF groups without changes in the control group. Hunger levels were moderate at baseline and decreased after 12 weeks intervention in all groups. There was a trend towards increased satisfaction in the ADF and TRF groups that was not statistically significant compared to the control group.

**Table 2:** Energy intake, hunger, satisfaction and fullness during the 12-week study

		Week 4	Week 12	*P*-value	*P*-value	Change	*P*-value
Feed day energy intake (kcal/d)	Control(n = 79)	1337 ± 108	1309 ± 167	0.874	0.581	−28 ± 137	0.575
	ADF (n = 90)	1356 ± 193	1327 ± 207	0.793		−29 ± 109	
	TRF(n = 95)	1374 ± 146	1358 ± 213	0.934		−16 ± 90	
Fast day energy intake (kcal/d)	ADF (n = 90)	324 ± 23	330 ± 31	0.677		12 ± 7	
Hunger	Control(n = 79)	25 ± 3	11 ± 2	0.013	0.819	−14 ± 1	0.943
	ADF (n = 90)	30 ± 4	14 ± 2	0.009		−16 ± 1	
	TRF(n = 95)	28 ± 3	13 ± 2	0.013		−15 ± 1	
Fullness	Control(n = 79)	8 ± 1	16 ± 1	0.119	0.778	8 ± 1	0.551
	ADF (n = 90)	4 ± 1	19 ± 1	0.001		15 ± 1	
	TRF(n = 95)	3 ± 1	17 ± 1	0.001		14 ± 1	
Satisfaction	Control(n = 79)	23 ± 4	28 ± 5	0.496	0.878	4 ± 1	0.640
	ADF (n = 90)	27 ± 6	35 ± 4	0.272		8 ± 2	
	TRF(n = 95)	26 ± 5	32 ± 4	0.431		6 ± 1	

### Body weight and body composition

Changes in body weight and body composition are shown in Table 3. After 4 weeks of dietary intervention, the body weight significantly decreased in the ADF group by 4.56 ± 0.41 kg (6.1 ± 0.5%) and in the TRF group by 3.62 ± 0.65 kg (4.83 ± 0.9%) relative to the control group (− 2.24 ± 0.34 kg; 3.07 ± 0.5%). After 12 weeks, the body weight further decreased significantly in the ADF group (− 4.04 ± 0.54 kg; 5.4 ± 0.7%) and the TRF group (3.25 ± 0.67 kg; 4.3 ± 0.9%) compared to the controls (− 1.85 ± 0.65 kg; 2.54 ± 0.9%). The ADF and TRF groups did not significantly differ at both timepoints.

**Table 3:** Body weight and risk factors for metabolic disease changes during the 12-week study

	Control(n = 79)		ADF(n = 90)		TRF(n = 95)		*P*-value	
	Week 4	Week 12	Week 4	Week 12	Week 4	Week 12	Week 4	Week 12
Body weight (kg)	70.32 ± 6.75	71.77 ± 6.90	70.76 ± 7.76	71.28 ± 7.02	71.31 ± 7.04	71.67 ± 7.37	0.648	0.709
BMI	26.48 ± 1.60	26.64 ± 1.66	25.97 ± 1.75	26.15 ± 1.58	26.48 ± 1.38	26.60 ± 1.46	0.049	0.071
Fat mass (kg)	27.91 ± 3.53	28.01 ± 3.49	27.09 ± 2.52	27.10 ± 2.52	27.36 ± 3.44	27.65 ± 3.34	0.241	0.165
Lean mass (kg)	42.41 ± 7.28	42.71 ± 7.67	43.67 ± 7.61	44.17 ± 7.79	43.96 ± 7.18	44.04 ± 7.47	0.352	0.395
Waist circumference	88.29 ± 4.73	88.54 ± 5.10	88.99 ± 5.20	87.19 ± 4.88	89.70 ± 7.42	87.58 ± 4.39	0.590	0.174
Cholesterol (mg/dL)	4.45 ± 1.36	4.65 ± 1.36	3.95 ± 1.08	4.15 ± 1.06	4.03 ± 1.55	4.37 ± 1.53	0.039	0.055
Total Direct HDL(mg/dL)	1.29 ± 0.55	1.18 ± 0.51	1.28 ± 0.41	1.17 ± 0.38	1.18 ± 0.47	1.15 ± 0.45	0.206	0.920
LDL (mg/dL)	2.55 ± 0.79	2.51 ± 0.76	2.82 ± 0.85	2.79 ± 0.83	2.73 ± 0.88	2.71 ± 0.87	0.101	0.085
Triglycerides (mg/dL)	2.28 ± 1.70	2.40 ± 1.70	2.21 ± 1.91	2.12 ± 1.90	2.27 ± 1.75	2.31 ± 1.75	0.965	0.582
Glucose (mg/dL)	5.18 ± 0.072	4.94 ± 1.22	5.36 ± 1.32	5.19 ± 0.67	5.21 ± 1.06	5.05 ± 1.35	0.500	0.355
Liver stiffness (kpa)	18.39 ± 4.64	18.36 ± 3.84	18.03 ± 2.25	18.05 ± 2.84	18.13 ± 4.11	18.37 ± 3.65	0.825	0.766

**Fig. 2:**
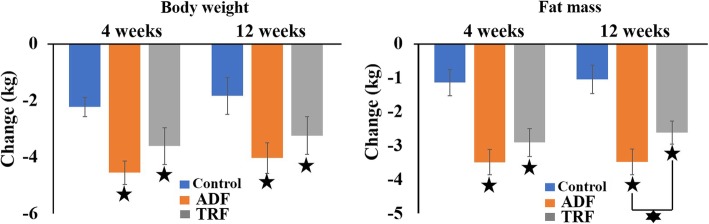
Changes in body weight and body composition at week 4 and week 12. Values are reported as mean ± standard deviation. ADF: alternate-day fasting; TRF: time-restricted feeding. Significant decreases in body weight and fat mass were observed in the ADF and TRF groups compared to the control group. : *P* < 0.001; : *P* < 0.05

After 4 weeks of intervention, fat mass was significantly (*P* < 0.001) reduced by 3.49 ± 0.37 kg (11 ± 1.2%) in the ADF group and by 2.91 ± 0.41 kg (9.6 ± 1.3%) in the TRF groups compared to the control group, with no differences observed between the ADF and TRF groups. In the ADF group a further significant (*P* < 0.001) reduction in fat mass by 3.48 ± 0.38 kg (11 ± 1.2%) was achieved after 12 weeks on the diet compared to the TRF group (− 2.62 ± 0.34 kg; 8.6 ± 1.1%) and the control group (− 1.05 ± 0.45 kg; 3.6 ± 0.93%). Fat-free mass did not change in any of the groups (Fig. 2).

### Risk factors for metabolic disease

**Fig. 3:**
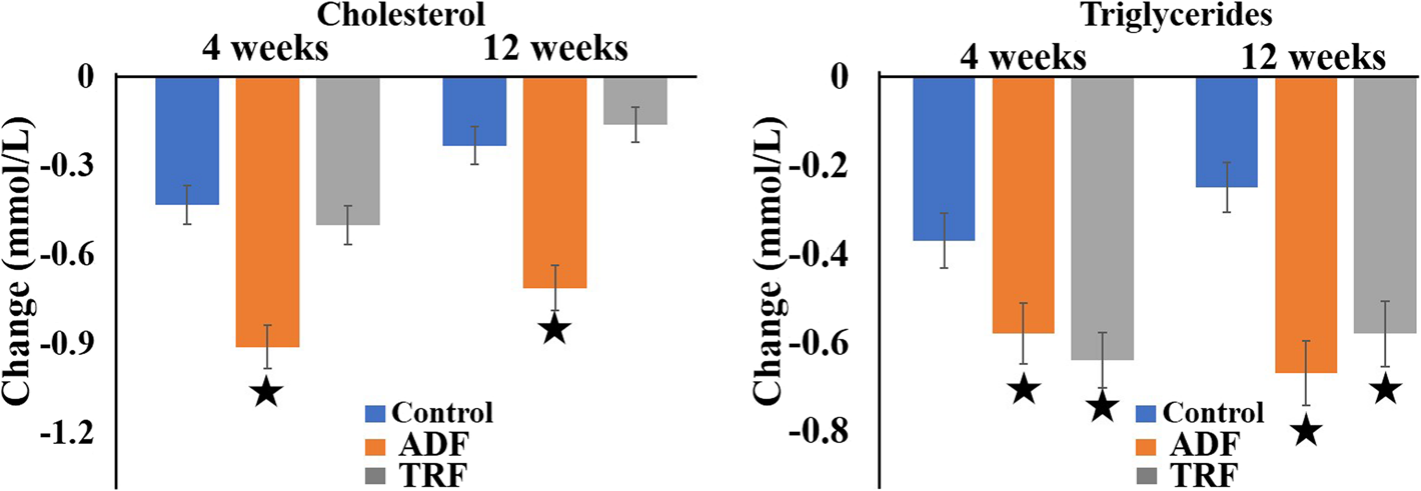
Changes in dyslipidaemia at week 4 and week 12. Values are reported as mean ± SEM (standard error of the mean) ADF achieved a significant decrease in total cholesterol compared to both the control and TRF groups. Triglycerides were significantly decreased in both the ADF and TRF groups compared to the control group. : P < 0.001; : *P* < 0.05.

Observed changes in plasma dyslipidaemia are shown in Table 3. Compared with baseline, there were no significant differences in total cholesterol, HDL, LDL and triglycerides among the 3 groups. Total cholesterol decreased significantly in the ADF group (*P* < 0.001) by 0.91 ± 0.07 mmol/L (18.5 ± 1.5%) at 4 weeks and by 0.71 ± 0.08 mmol/L (14.5 ± 1.6%) at 12 weeks compared to the control and TRF groups. After 4 weeks, Triglycerides were also significantly (P < 0.001) decreased in the ADF group by 0.58 ± 0.07 mmol/L (22 ± 1.8%) and in the TRF group by 0.67 ± 0.07 mmol/L (25 ± 2.0%) relative to the control group (− 0.37 ± 0.06 mmol/L; 14 ± 1.2%). After 12 weeks, Triglycerides further significantly decreased in ADF group (0.64 ± 0.06 mmol/L; 25 ± 1.9%) and TRF (0.58 ± 0.07 mmol/L; 20 ± 1.7%) compared to the controls (− 0.25 ± 0.06 mmol/L; 9 ± 1.0%) (Fig. 3). No other within- or between-group differences were observed in changes in fasting insulin, glucose, and systolic or diastolic blood pressure at both time points (data not shown).

## Discussion

This study is the first single-centre large-scale randomised trial to examine the impact of ADF on NAFLD patients. Our study showed that ADF was a safe and tolerable diet regimen for NAFLD patients and led to reductions in body weight, fat mass, total cholesterol, and triglycerides.

The primary goal of this study was to determine whether NAFLD patients could benefit from ADF. Lifestyle changes are typically the first-line therapy of NAFLD, as diet and exercise enhance weight loss and improve steatosis and lobular inflammation. Dietary composition is an important point to consider as replacing dietary fats with carbohydrates can reduce intrahepatic lipid content [4, 5]. Previous studies on NAFLD have demonstrated that a weight loss of at least 5% of body weight is associated with improvements in steatosis and insulin resistance, while a weight loss of at least 7–9% is apparently required to alleviate inflammation and histological disease activity [22, 23]. IF typically results in a modest weight loss (2–10%) when administered over a period of 2–12 weeks. TRF has been shown to significantly decrease the body mass index and has been identified as a potential cost-effective intervention in obese and NAFLD patients in a previous study [24]. Nonetheless, the relative weight loss in this study was < 5% and it was acknowledged that the effects of TRF on NAFLD patients deserves further study [25]. The main finding of our study is that NAFLD patients may indeed benefit from ADF, with an average weight loss of 6.1% observed after 4 weeks of dietary intervention and a further 5.4% weight loss after 12 weeks. Importantly, this loss in body weight was primarily due to a loss in fat mass with no changes in the fat-free mass observed. These changes in body composition tended to be more pronounced in the ADF group compared to the other groups. A higher weight loss was induced by ADF than by TRF; we speculate that this difference can be attributed to a greater overall caloric restriction in the ADF group, as subjects in the TRF group were not required to monitor their calorie intake.

We observed that dietary adherence was very high during 12 weeks (97.5%) and that the participants in the ADF group adjusted well to the fast day protocol. Five subjects in the ADF group and 2 subjects in the TRF group dropped out of the trial due to an inability to adhere to the diet, but the overall dropout rate was still below 10% with no significant differences between the 3 groups. In agreement with previous reports, subjects in the ADF group experienced no or only mild hyperphagic response on the feed day in response to the lack of food on the previous fast day [15, 26]. The lack of hyperphagia allowed for an overall high energy restriction throughout the study and undoubtedly contributed to the sizeable degree of weight loss observed. In terms of eating behaviours, dietary satisfaction was moderate at baseline and had not changed by week 12 in any of the groups. Nonetheless, the perceived feeling of hunger decreased and the feeling of fullness increased during the study period relative to the baseline assessment, which may contribute to a long-term adherence to such a diet. Neither ADF nor TRF appeared to increase eating disorder symptoms such as depression, binge eating, purgative behaviour, fear of being overweight, or avoidance of forbidden foods, and may even lead to beneficial effects on body image perception.

NAFLD is frequently associated with dyslipidaemia (high triglycerides, low HDL, high VLDL) and increased levels of pro-inflammatory cytokines which are atherogenic and promote the development of cardiovascular disease [2]. Potential beneficial effects of IF on dyslipidaemia are controversial [13, 27, 28]. Cholesterol, total direct HDL, LDL, and triglycerides have been shown to be reduced by a fasting diet, but these effects were not statistically different from the baseline values. In our study, total cholesterol and triglycerides were significantly decreased in the ADF group after 4 and 12 weeks compared to the control group, while only triglycerides were significantly reduced in the TRF group. Accumulating evidence suggests that a weight loss > 5% is required to improve plasma lipid concentrations and glucoregulatory factors, therefore the weight loss achieved by the TRF and control diets were probably not large enough to exert any effect on dyslipidaemia. Changes in other risk factors of metabolic disease did not differ between the groups, suggesting that the diets had similar effects on these parameters. These results agree with previous studies suggesting that ADF causes improvements in fasting lipids [13, 16].

Certain limitations of this study should be addressed. First, although ADF was not associated with greater weight regain after longer term follow-up [12], the study period of 12 weeks was relatively short. Longer trials are required to determine the degree of weight loss that can be achieved, and whether liver stiffness could be reversed with IF diets such as ADF and TRF. Second, we did not assess physical activity throughout the trial and thus any effect of an increased energy expenditure on weight loss remains unknown. Third, adherence and dietary intake were self-reported; therefore, our estimates on eating duration and caloric deficit may be inaccurate. This issue could be circumvented by implementing mobile apps to assess adherence to the prescribed eating window in real-time.

## Conclusions

In summary, our results suggest that ADF is a safe and tolerable diet regimen for patients with NAFLD with a moderate caloric restriction that can achieve an effective weight loss of > 5% within a relatively short period of time (4 weeks). Future studies should employ objective measures of energy intake, sleep duration, energy expenditure, and markers of metabolic disease and comprise a more diverse study population.

## Data Availability

The datasets generated during the current study are available from the corresponding author on reasonable request.

## Abbreviations

ADF: Alternate-day fasting
HDL: High density lipoprotein
IF: Intermittent fasting
IR: Insulin resistance
LDL: Low density lipoprotein
NAFLD: non-alcoholic fatty liver disease
T2DM: type 2 diabetes mellitus
TRF: Time-restricted feeding
